# A rare case of renal AHL amyloidosis with marked complement deposition: clinicopathologic and proteomic findings

**DOI:** 10.1186/s12882-026-05017-6

**Published:** 2026-04-30

**Authors:** Yoshihiro Yamamoto, Takahiro Shinzato, Shusuke Shishihara, Ken Matsuo, Arimi Ishikawa, Naomi Kuwahara, Akira Shimizu, Kojiro Nagai

**Affiliations:** 1 Department of Nephrology, Shizuoka General Hospital, 4-27-1 Kita-Ando, Aoi-ku, Shizuoka, 420-8527 Japan; 2 Department of Analytic Human Pathology, Nippon Medical School, Tokyo, Japan

**Keywords:** AHL amyloidosis, Monoclonal gammopathy of renal significance (MGRS), Renal complement deposition, Fibrillary glomerulonephritis (FGN), Dna J heat-shock protein family B member 9 (DNAJB9), Laser microdissection mass spectrometry (LMD/MS)

## Abstract

**Background:**

Amyloidosis comprises a heterogeneous group of diseases characterized by extracellular deposition of β-pleated-sheet fibrillar proteins that cause progressive organ dysfunction. Among immunoglobulin-related amyloidosis, combined heavy- and light-chain (AHL) amyloidosis is extremely rare, which accounts for approximately 7%. Several studies have demonstrated that complement components can be detected in renal amyloidosis. We report a rare case of renal AHL amyloidosis with marked complement deposition with clinicopathologic and proteomic insights.

**Case presentation:**

A 76-year-old woman with a five-year history of microscopic hematuria developed mild renal dysfunction (serum creatinine 1.05 mg/dL) and proteinuria (0.95 g/day). Physical and serologic evaluations showed no evidence of systemic amyloidosis or autoimmune disease. Serum and urine immunofixation detected an IgG-κ M-protein, and bone marrow findings were consistent with monoclonal gammopathy of undetermined significance. Kidney biopsy demonstrated Congo red–positive fibrillar deposits with IgG1, κ, and complement (C3/C1q) staining. Electron microscopy revealed non-branching fibrils measuring 8 to 12 nm in diameter. Because marked complement deposition was observed, fibrillary glomerulonephritis (FGN) was considered in the differential diagnosis. However, negative Dna J heat-shock protein family B member 9 immunostaining raised suspicion for amyloidosis. Mass spectrometry identified IgG1 heavy and κ light chains together with serum amyloid P and apolipoprotein E, confirming IgG1-κ-type AHL amyloidosis.

**Conclusions:**

This case illustrates a presentation of AHL amyloidosis with marked complement deposition. Recent study reveals that complement components can be detected in a subset of amyloidosis cases. These features can complicate the differential diagnosis from FGN and highlight the importance of an integrated diagnostic approach combining histopathology, immunostaining, and proteomic analysis. Furthermore, clone-directed therapy targeting the pathogenic plasma cell clone may represent a rational therapeutic strategy for monoclonal immunoglobulin–associated renal disease.

**Supplementary Information:**

The online version contains supplementary material available at 10.1186/s12882-026-05017-6.

## Background

Amyloidosis comprises a heterogeneous group of diseases characterized by extracellular deposition of fibrillar proteins with a β-pleated-sheet configuration. These disorders arise from abnormal folding and aggregation of precursor proteins, which form amyloid fibrils and lead to progressive organ dysfunction [1, 2].

To date, 42 amyloidogenic precursor proteins have been identified: 14 associated with systemic amyloidosis, 24 with localized forms, and 4 linked to both [1, 2]. The International Society of Amyloidosis (ISA) classifies renal amyloidosis into 14 subtypes, whereas a large proteomic cohort study in the United States identified 11 distinct renal amyloid types using mass spectrometry [3]. Renal AL amyloidosis accounted for 75–80%, followed by AA (7–14%), Leukocyte chemotactic factor 2 -associated amyloidosis (3–4%), heavy chain amyloidosis and heavy and light chain amyloidosis (5%); the remaining types each represented less than 1% [4]. In the ISA amyloid fibril protein nomenclature, immunoglobulin-derived amyloidosis is classified as AL or AH based on the precursor protein [1]. The diagnosis of AHL requires intense staining of 1 heavy chain and 1 light chain [2].

Within the spectrum of monoclonal gammopathy of renal significance (MGRS), disorders characterized by fibrillar deposits include AL/AH/AHL amyloidosis and fibrillary glomerulonephritis (FGN). AL/AH/AHL amyloidosis typically demonstrates positive Congo red staining and monotypic deposition of immunoglobulin heavy and/or light chains [5–7]. By contrast, FGN usually exhibits polytypic IgG deposits with both κ and λ light chains on immunofluorescence [8, 9]. Although complement deposition has been described as negative, minimal, or weak in some reports about renal amyloidosis [6, 10–12], several studies have recently demonstrated that complement components can be detected in a subset of cases. Yao et al. reported that approximately one-third of patients with renal AL amyloidosis showed co-deposition of immune complexes and complement in glomeruli [13]. In addition, proteomic analyses have identified multiple complement pathway proteins within amyloid deposits, including C9 and other complement components [14, 15]. Large-scale proteomic studies further suggest that complement pathway proteins are enriched in many amyloid types, indicating that complement activation can happen in renal amyloidosis [16]. In contrast, complement deposition is commonly reported in FGN [8, 9]. In the present case, because of Congo red positivity, complement deposition, and the presence of fibrillar structures, congophilic FGN was considered; however, Dna J heat-shock protein family B member 9 (DNAJB9) immunostaining was negative. The presence of monoclonal deposition of both heavy and light chains suggested AHL amyloidosis. Mass spectrometry was performed to confirm this rare subtype of amyloidosis. The deposits were negative for DNAJB9 and serum amyloid A (SAA) but positive for amyloid signature proteins (serum amyloid P (SAP) and apolipoprotein E (ApoE)), as well as IgG1 and κ, both of which showed monotypic staining on immunofluorescence. These findings supported a diagnosis of IgG1κ-type AHL amyloidosis.

## Case presentation

### Clinical presentation and laboratory findings

Five years before renal biopsy, the patient had normal renal function, with a serum creatinine (Cr) level of 0.71 mg/dL and microscopic hematuria without proteinuria. Two years before biopsy, creatinine increased to 0.85 mg/dL and was accompanied by mild urinary abnormalities. Creatinine subsequently increased to 1.12 mg/dL, with worsening proteinuria and hematuria, prompting referral for further evaluation and admission for renal biopsy. Her past medical history included left ear’s hearing loss at age 60 and hypertension and dyslipidemia at 66. On physical examination, height was 142.5 cm and weight was 33.2 kg (BMI 16.3 kg/m²). Body temperature was 37.2 °C, pulse 90 bpm, blood pressure 140/74 mmHg, and oxygen saturation 96% on room air. The patient was underweight. A detailed review of the patient’s remote history revealed no significant weight loss over the past five years. There was no anemia, jaundice, lymphadenopathy, macroglossia, or peripheral edema. Cardiopulmonary, abdominal, and neurologic examinations were unremarkable.

Electrocardiography revealed normal sinus rhythm without low voltage. Chest radiography showed a cardiothoracic ratio of 47% without pleural effusion. Abdominal ultrasonography demonstrated mildly atrophic kidneys (right 8.7 cm, left 9.2 cm) with slightly increased cortical echogenicity. Echocardiography showed preserved ejection fraction (72%) without wall thickening or valvular abnormalities.

Laboratory findings on admission included a white blood cell count of 3,500/µL, hemoglobin 10.3 g/dL, and platelet count 303,000/µL. Serum total protein was 7.4 g/dL, albumin 3.8 g/dL, blood urea nitrogen 19 mg/dL, creatinine 1.05 mg/dL, C-reactive protein 0.02 mg/dL, BNP 15.5 pg/mL, and creatinine-based eGFR 41 ml/min/1.73m^2^. Because cystatin C was not measured, renal function was assessed using creatinine-based eGFR which may have overestimated the patient’s renal function due to low BMI.

Immunologic testing revealed IgG 1,252 mg/dL, IgA 16 mg/dL, and IgM 13 mg/dL, with normal complement levels (C3 105 mg/dL, C4 23 mg/dL, CH50 56 U/mL). Autoantibodies and viral serologies were negative.

Urinalysis revealed protein (2+) and blood (2+), with 5 to 9 red blood cells per high-power field of glomerular origin. Twenty-four-hour urinary protein excretion was 0.95 g/day, and the urine protein-to-creatinine ratio was 2.3 g/gCr. Twenty-four-hour urinary albumin excretion was 0.64 g/day, and the urine albumin-to-creatinine ratio was 1.9 g/gCr. Because of the patient’s low BMI and presumed low muscle mass, serum creatinine–based estimates of urinary excretion may have been influenced by reduced creatinine generation. Laboratory findings on admission are summarized in Table 1. Serum and urine immunofixation electrophoresis identified an IgG-κ M-protein (Supplementary FIgure 1) with a normal free light chain ratio (κ/λ = 1.47). Bone marrow biopsy demonstrated 5 to 10% plasma cells positive for CD138, IgG, and κ, and negative for IgA, IgM, and λ. These findings supported a diagnosis of IgG-κ monoclonal gammopathy of undetermined significance. Given the progressive renal dysfunction and increasing proteinuria in the setting of monoclonal gammopathy, a kidney biopsy was performed to determine the underlying renal pathology and evaluate the possibility of monoclonal gammopathy–associated kidney disease.

**Table 1:** Laboratory data of the patient

Parameter	Value	Reference range
White blood cell count	3,500 /µL	3,300–8,600
Hemoglobin	10.3 g/dL	11.6–15.0
Platelet count	303,000 /µL	158,000–348,000
Total protein	7.4 g/dL	6.6–8.1
Albumin	3.8 g/dL	4.1–5.1
Blood urea nitrogen	19 mg/dL	8–20
Creatinine	1.05 mg/dL	0.46–0.79
C-reactive protein	0.02 mg/dL	< 0.14
BNP	15.5 pg/mL	< 18.4
IgG	1252 mg/dL	861–1747
IgA	16 mg/dL	93–393
IgM	13 mg/dL	16–269
Complement C3	105 mg/dL	73–138
Complement C4	23 mg/dL	11–31
CH50	56 U/mL	30–60
Proteinuria (24-h urine)	0.95 g/day	< 0.15
Urine protein–creatinine ratio	2.32 g/gCr	< 0.15
Hematuria	5–9 RBC/HPF	< 3
Free light chain ratio (κ/λ)	1.47	0.26–1.65

BNP: B-type natriuretic peptide, CH50: 50% hemolytic complement activity

### Renal pathological findings

Light microscopy showed that, among 16 sampled glomeruli, two were globally sclerosed.

**Fig. 1:**
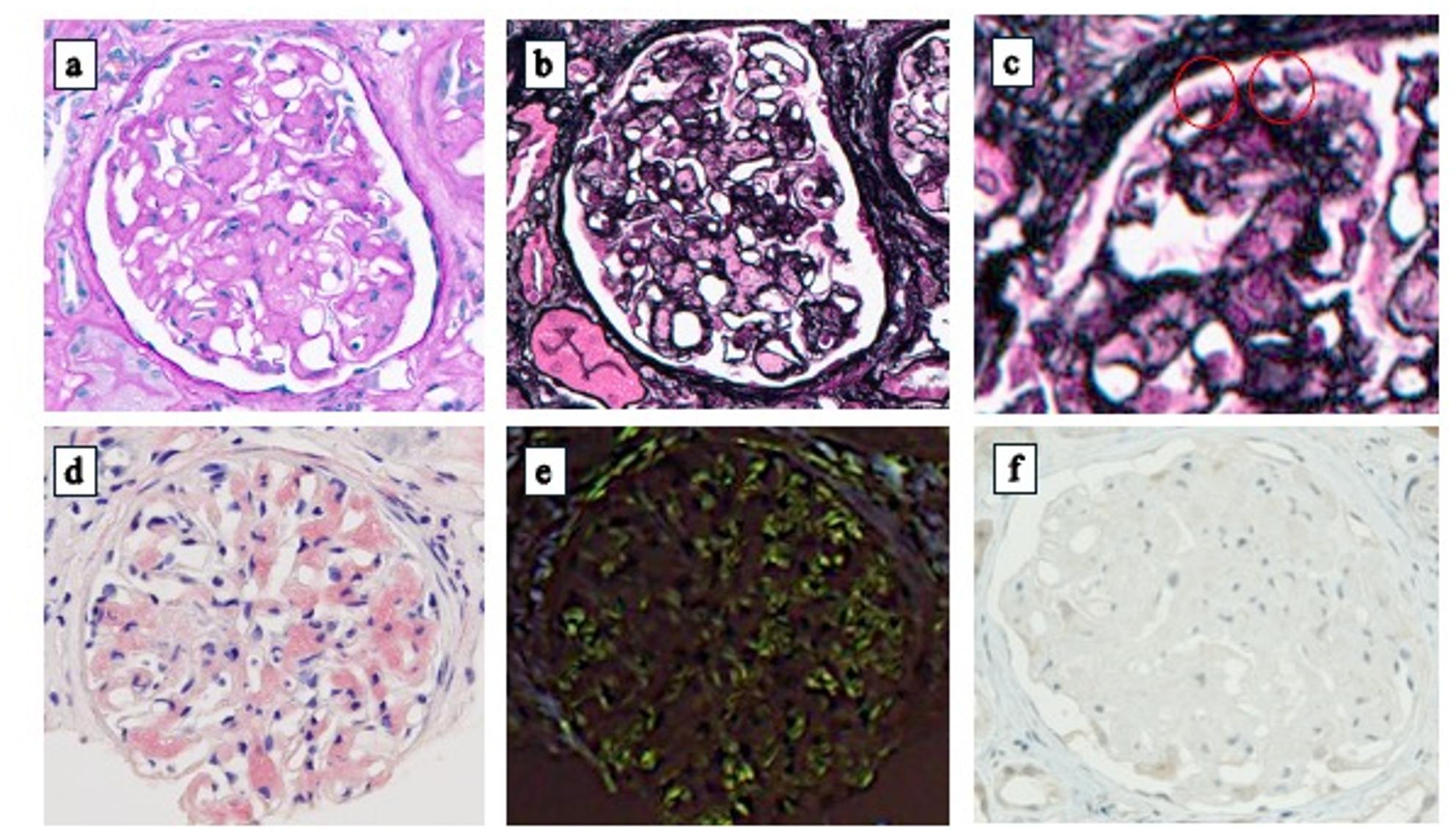
Renal pathological findings. (**a**) periodic acid–Schiff staining; (**b**) periodic acid–methenamine silver staining; (**c**) high-power view of spicule formation in red circle; (**d**) Congo red staining; (**e**) apple-green birefringence under polarized light in glomeruli; (**f**) Dna J heat-shock protein family B member 9 staining

Mesangial expansion was evident, with amorphous periodic acid–Schiff positive and periodic acid–methenamine silver negative deposits in the mesangium, accompanied by spicule formation (Fig. 1a–c). Congo red staining was positive and showed characteristic apple-green birefringence under polarized light (Fig. 1d, e). Congo red staining demonstrated amyloid deposits in all three renal compartments (glomeruli, vascular walls, and tubulointerstitium). To provide a more quantitative assessment of amyloid deposition, we evaluated the distribution of amyloid using the amyloid load scoring system proposed by Rubinstein et al. [17]. Based on this scoring system, the amyloid load was graded as follows: glomeruli: score 2, tubulointerstitium: score 1, and vessels: score 1. DNAJB9 staining was negative (Fig. 1f).

**Fig. 2:**
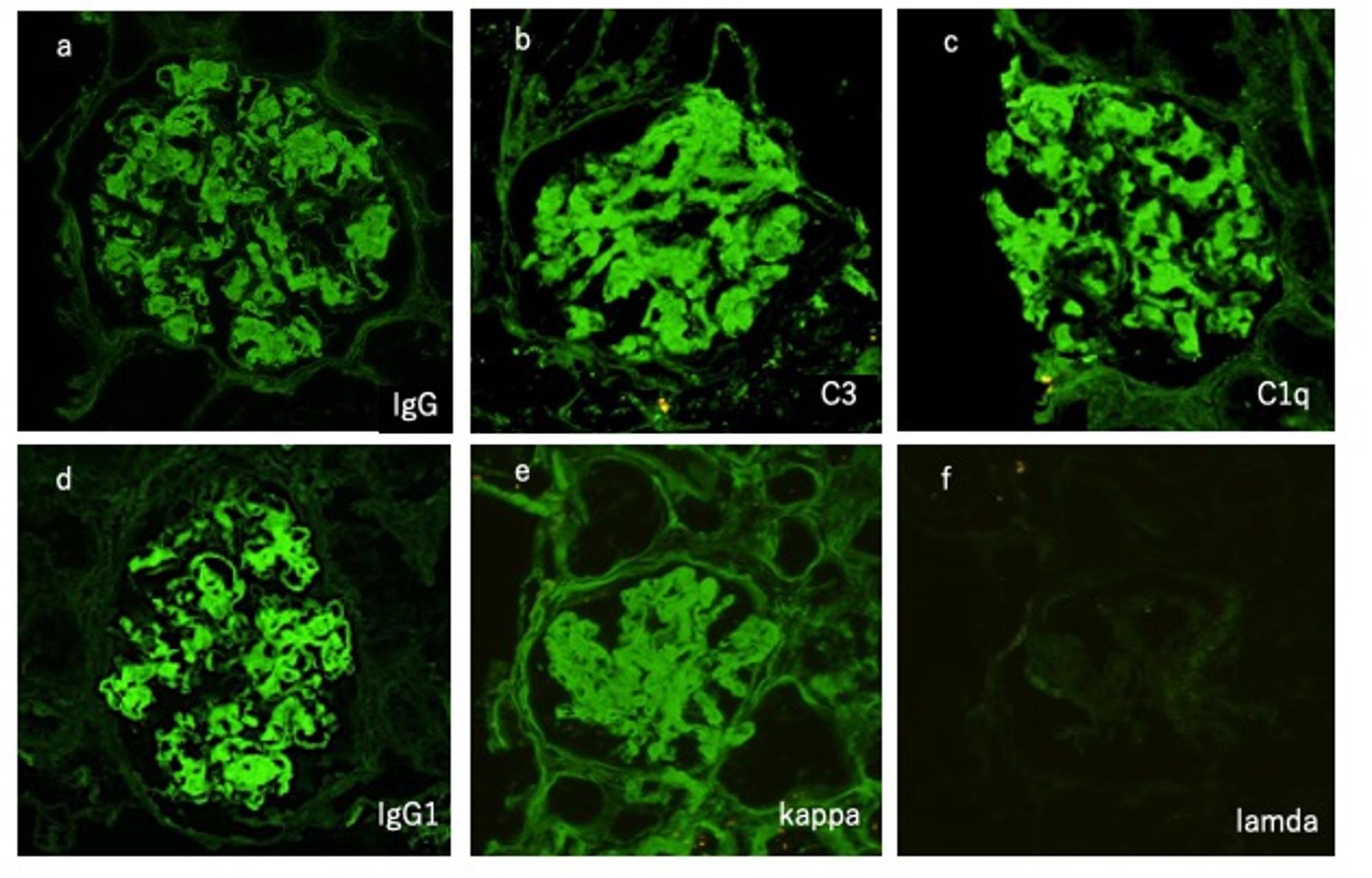
Immunofluorescence staining showing positivity for IgG, IgG1, C3, C1q, kappa. (**a**) IgG; (**b**) C3; (**c**) C1q; (**d**) IgG1; (**e**) kappa; (**f**) lamda

Immunofluorescence microscopy demonstrated IgG (2+), C3 (2+), C1q (2+), and κ light chain (2+) deposition, whereas λ light chain was absent. IgG subclass analysis revealed predominant IgG1 (2+) staining, with negative staining for IgG2, IgG3, and IgG4 (Fig. 2). Regarding the distribution of immunofluorescent staining, both immunoglobulin and complement staining were weak in the vascular and interstitial compartments, without a clear difference in intensity between the two, whereas the most evident staining was observed in the glomeruli.

**Fig. 3:**
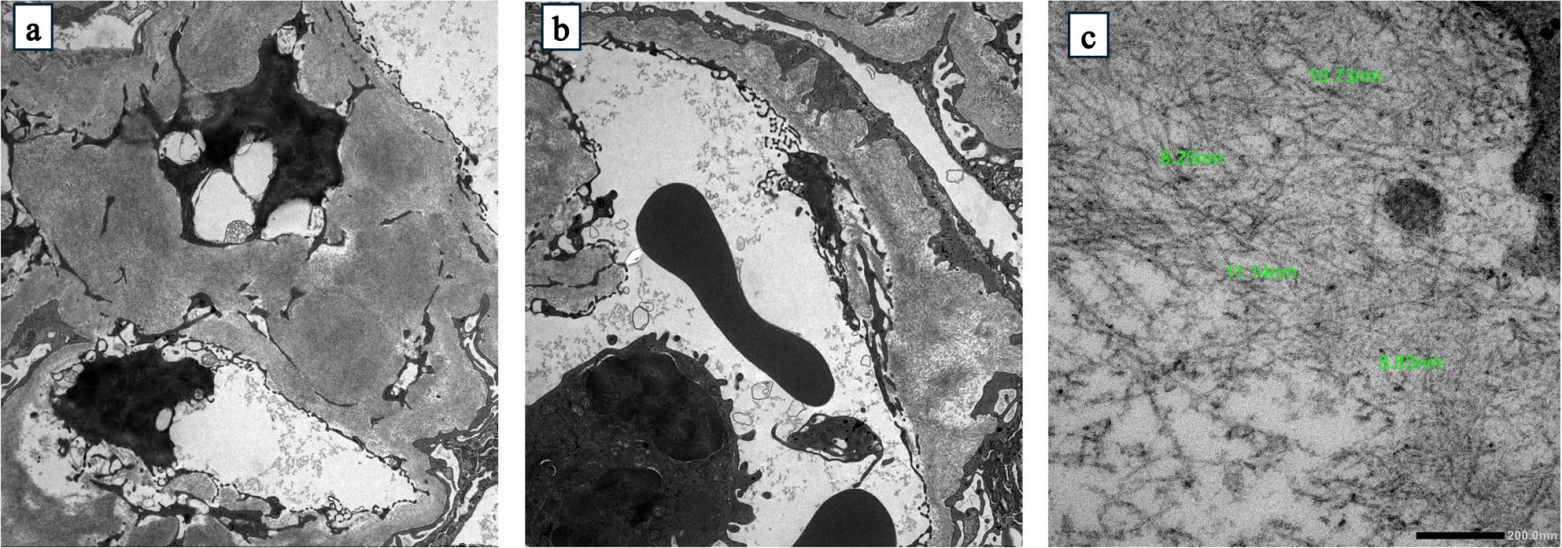
Electron micrograph showing fibrillary deposits measuring 8 to 12 nm in diameter within the mesangial (**a**), glomerular basement membrane (**b**,** c**)

Electron microscopy identified randomly oriented, non-branching fibrils measuring 8 to 12 nm in diameter within the mesangial, glomerular basement membrane (Fig. 3), consistent with amyloid fibril deposition.

### Laser microdissection (LMD)–liquid chromatography-tandem mass spectrometry (LC–MS/MS)

**Fig. 4:**
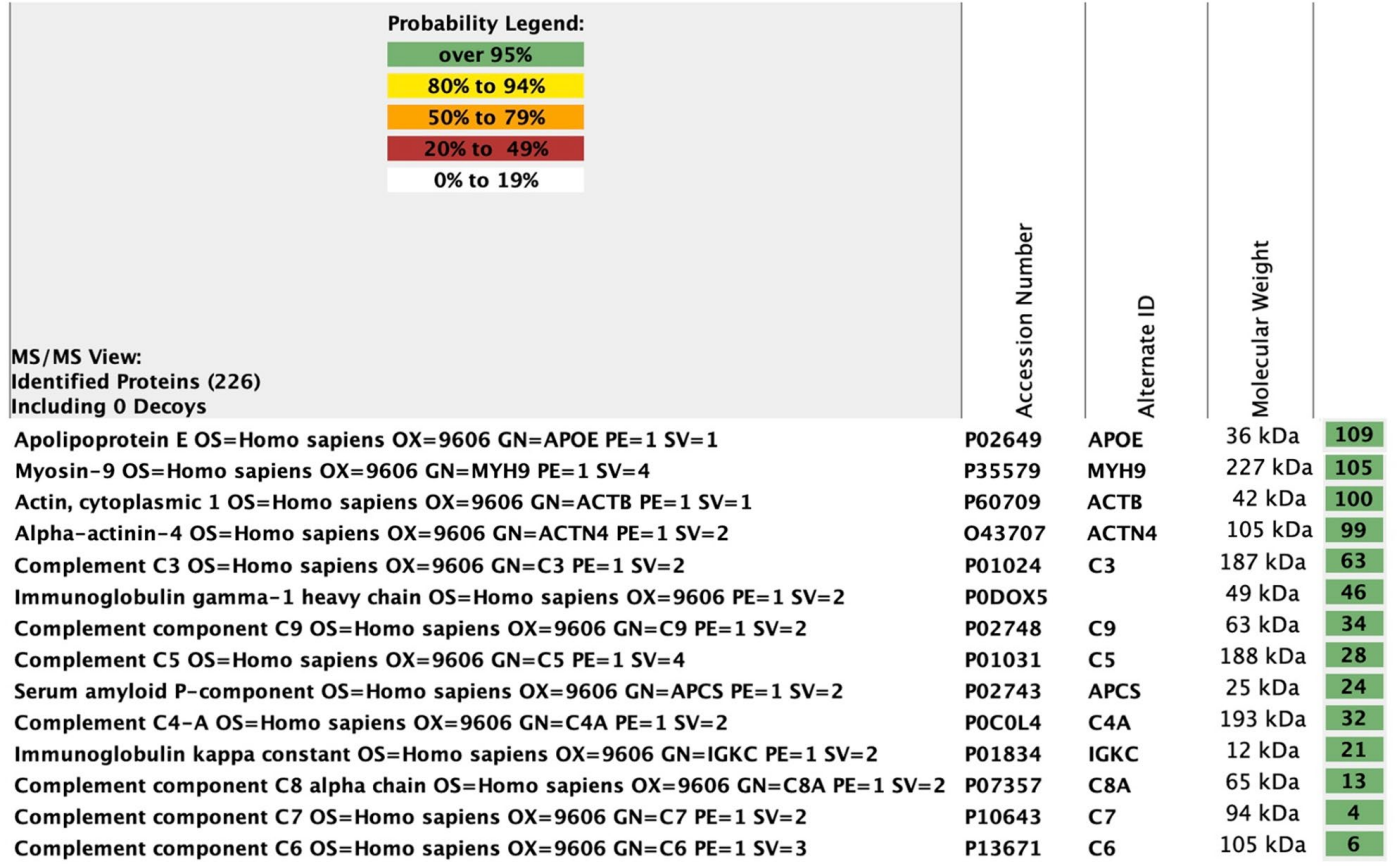
LMD/MS results based on the Scaffold database. Amyloid proteins (immunoglobulin gamma-1 heavy chain and immunoglobulin κ light chain), amlyloid-related proteins (serum amyloid P and apolipoprotein E), and complement proteins (complement 3–9) were detected

LMD–LC–MS/MS analysis was performed on Congo red–positive glomerular areas to define the protein composition of the deposits. Proteomic profiling detected 46 spectra corresponding to the IgG1 heavy chain and 24 spectra corresponding to the κ light chain, together with the amyloid signature proteins SAP and ApoE. DNAJB9, a specific biomarker for FGN, was not identified. In addition, proteomic profiling detected complement components including C3 (63 spectra), C4-A (32 spectra), C9 (34 spectra), C5 (28 spectra), C8 (13 spectra), C7 (4 spectra), and C6 (6 spectra). (Fig. 4). These proteomic findings, combined with the histopathologic and immunofluorescence results, established the final diagnosis of IgG1-κ-type AHL amyloidosis.

### Clinical course

**Fig. 5:**
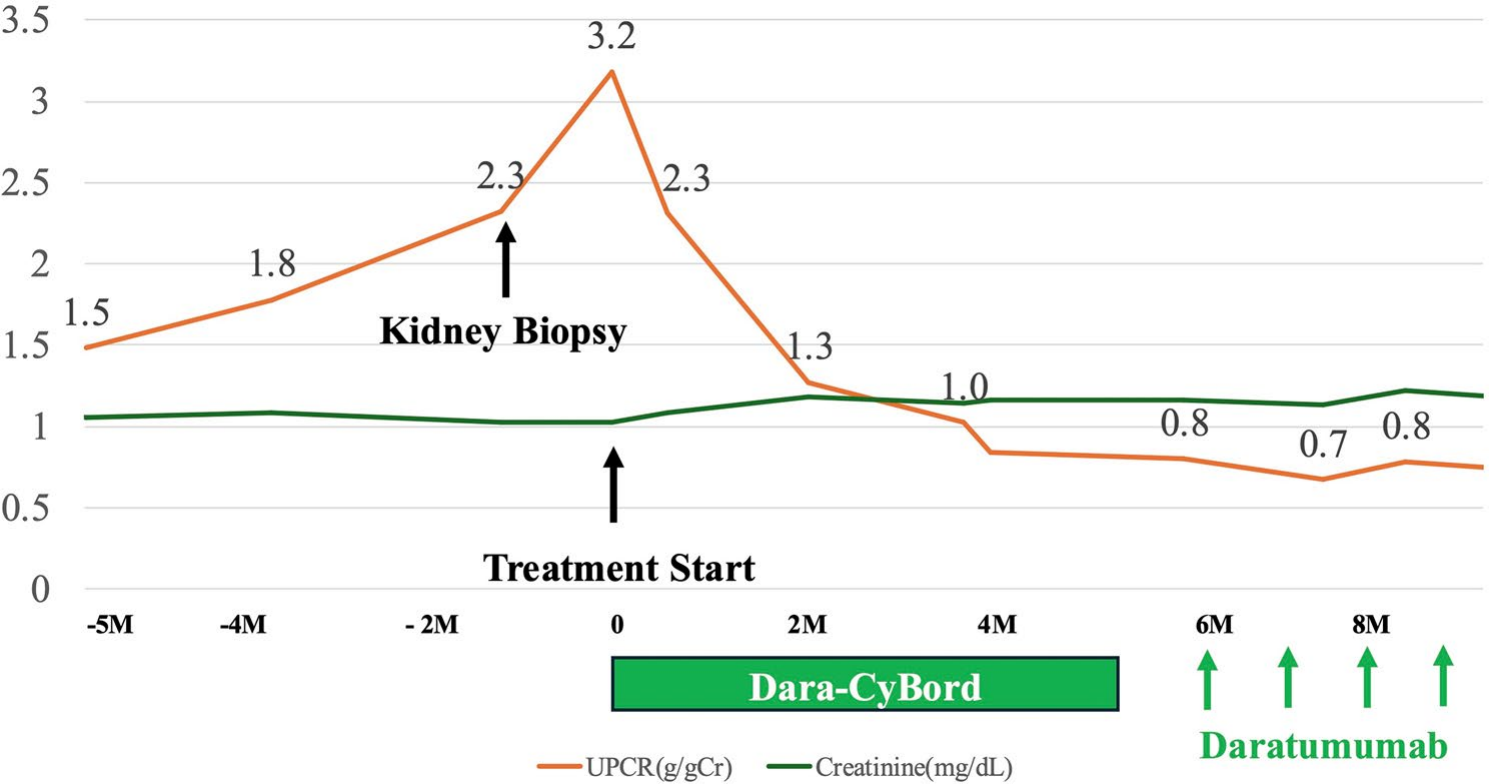
Clinical course of the patient. Dara-CyBord; daratumumab, cyclophosphamide, bortezomib, and dexamethasone. UPCR; urine protein-to-creatinine ratio

Treatment was initiated with daratumumab (1,800 mg/week), cyclophosphamide (200 mg/week), bortezomib (1.3 mg/m²/week), and dexamethasone (20 mg/week), followed by monthly daratumumab (1,800 mg/month). After seven months of therapy, proteinuria decreased from 3.2 g/gCr to approximately 0.7 g/gCr, while estimated glomerular filtration rate (eGFR) remained stable. The patient’s clinical course is summarized in Fig. 5. This patient was followed for 10 months after treatment initiation. No significant infectious complications were observed.

## Discussion

MGRS refers to renal diseases caused by nephrotoxic monoclonal immunoglobulins produced by a small clonal B-cell or plasma cell population that does not meet hematologic criteria for multiple myeloma or lymphoma [18].

According to the International Kidney and Monoclonal Gammopathy Research Group, MGRS lesions are classified as deposition diseases (e.g., AL/AH/AHL amyloidosis, light chain deposition disease, heavy chain deposition disease) or proliferative lesions (e.g., proliferative glomerulonephritis with monoclonal immunoglobulin deposits, cryoglobulinemic glomerulonephritis, monoclonal immunotactoid glomerulopathy, intracapillary monoclonal IgM glomerulopathy, monoclonal membranous nephropathy, C3 glomerulopathy associated with monoclonal gammopathy), based on the pathogenic mechanism and pattern of immunoglobulin deposition [6, 18]. Most FGN cases demonstrate polyclonal IgG deposition with both κ and λ light chains, although monotypic variants have been reported in approximately 6% of cases [8].

Amyloidosis is Congo red positive, exhibits apple-green birefringence under polarized light, and contains fibrils measuring 8 to 12 nm in diameter. In contrast, FGN is characterized by randomly arranged fibrils measuring 15 to 25 nm in diameter and shows positive DNAJB9 staining, typically negative Congo red staining [8, 9]. In the present case, the fibril diameter of 8 to 12 nm with negative staining for DNAJB9 excludes the diagnosis FGN. In some FGN cases, fibrils measuring ≤ 10 nm have also been described. For example, Nasr et al. reported fibril diameters ranging from 10 to 30 nm in FGN, and a multi-institutional cohort study demonstrated a median fibril diameter of 15 nm with a range of 9–25 nm. Therefore, the ultrastructural distinction between amyloidosis and FGN based solely on fibril diameter may be difficult in certain cases [8, 9].

In addition, recently, a subset termed “congophilic FGN,” characterized by weak Congo red positivity, has been described, further complicating diagnostic differentiation. Notably, up to 35% of congophilic FGN cases are associated with monoclonal gammopathy and may be misdiagnosed as amyloidosis, potentially leading to inappropriate chemotherapy [18]. Therefore, accurate discrimination between amyloidosis and FGN requires molecular analysis. DNAJB9 is a highly specific biomarker for FGN, independent of Congo red positivity [19, 20]. Conversely, amyloidosis consistently demonstrates amyloid signature proteins, including SAP and ApoE, on mass spectrometry, reflecting their roles in fibrillogenesis [21]. Diagnostic criteria include identification of a heavy-chain and/or light-chain isotype together with at least two amyloid signature proteins (SAP, ApoE, apolipoprotein A-IV) [7]. In this case, DNAJB9 was absent, whereas SAP and ApoE were detected, confirming amyloidosis (Fig. 4).

Said et al. analyzed 474 renal amyloidosis cases and reported that AL, AH, and AHL amyloidosis collectively accounted for 85.9% of all cases [22]. LMD/MS was required in approximately 16% of cases, primarily because of limited tissue availability, negative κ or λ staining, equivocal or nonspecific immunofluorescence, or strong heavy-chain staining regardless of light-chain presence. The authors emphasized that immunofluorescence antibodies recognize epitopes within the constant regions of immunoglobulins; therefore, deletion or structural modification of these regions may result in false-negative staining. Moreover, nonspecific staining for multiple immunoglobulins or complement components may obscure the distinction between AA amyloidosis and AL/AH/AHL amyloidosis [6, 23]. In the present case, SAA immunostaining was not performed; however, mass spectrometry did not detect SAA peptides, thereby excluding AA amyloidosis (Fig. 4).

According to Said et al., AHL amyloidosis accounts for approximately 7% of immunoglobulin-related renal amyloidosis [22]. In LMD/MS analysis, spectra corresponding to the immunoglobulin heavy chain are as abundant as, or more abundant than, those of the light chain, and immunofluorescence demonstrates comparably intense staining for both [22]. In the present case, proteomic analysis of micro-dissected Congo red–positive deposits detected peptides derived from both the IgG1 heavy chain and the κ light chain. Specifically, 46 spectra corresponding to the IgG1 heavy chain and 24 spectra corresponding to the κ light chain were identified. The detection of both heavy- and light-chain–derived peptides with a higher number of spectra corresponding to the heavy chain than to the light chain, further supports the diagnosis of AHL amyloidosis (Fig. 4).

With respect to complement deposition, laser microdissection and proteomic analyses by Sethi et al. demonstrated frequent detection of classical and terminal complement pathway components (C3, C4, C9) in FGN, whereas AL amyloidosis rarely contains complement proteins [10]. In contrast, a large-scale proteomic study of 2,650 renal amyloidosis cases identified 58 AH and 73 AHL amyloidosis cases (1.4% and 1.7%, respectively). That study showed greater complement pathway enrichment in κ-type AL deposits than in λ-type deposits, and higher complement and extracellular matrix protein content in AH amyloidosis than in AL amyloidosis [16]. This case was IgG1-κ-type AHL amyloidosis, which may partly explain the presence of complement components detected in the proteomic analysis.

The article by Charalampous et al. also reported stage of renal amyloid affecting the type of protein expressed. In their series, stage 1 or 2 had increased cytoskeletal proteins and decreased complement activation / collagen deposition [16]. This case corresponded to stage 2 according to the staging system for renal outcome [24] and, mass spectrometry revealed relatively abundant cytoskeletal proteins, including MYH9, ACTB, and ACTN4 (Fig. 4).

ApoE is a well-recognized amyloid-associated protein frequently detected in proteomic analyses of amyloid deposits [25, 26]. Beyond its role in lipid metabolism, ApoE has been implicated in immune regulation and inflammatory responses. Recent proteomic studies have shown that ApoE can be enriched in dense deposits in C3 glomerulopathy, suggesting a potential interaction between apolipoproteins and complement-mediated renal injury [27].

In addition, complement components detected in renal tissue may reflect local intra-renal complement activation triggered by tissue injury rather than systemic complement dysregulation. Therefore, the coexistence of ApoE and complement components in the present case may represent secondary complement activation associated with tissue injury within the kidney.

Recent evidence indicates that IgG Fc galactosylation enhances C1q binding and complement activation, underscoring the structural dependence of Fc-mediated effector functions [28]. Some AHL amyloidosis cases therefore exhibit complement deposition and mesangial proliferative features, complicating differentiation from FGN.

Bortezomib-based regimens are widely used as first-line therapy for plasma cell–related amyloidosis. In recent years, daratumumab, a monoclonal antibody targeting CD38 expressed on plasma cells, has demonstrated significant efficacy in AL amyloidosis. In the phase III ANDROMEDA trial, the addition of daratumumab to cyclophosphamide, bortezomib, and dexamethasone significantly improved hematologic and organ response rates compared with CyBorD alone [29]. In this case, clone-directed therapy targeting the underlying plasma cell disorder was initiated. The patient was treated with cyclophosphamide, bortezomib, dexamethasone, and daratumumab. Although clinical trials have primarily focused on AL amyloidosis, similar therapeutic strategies may be applicable to other immunoglobulin-related amyloidoses, including AHL amyloidosis. (Fig. 5)

## Conclusion

This case highlights several important clinical implications. AHL amyloidosis is a rare subtype of immunoglobulin-related amyloidosis that may present with complement deposition. Such findings may complicate the differential diagnosis from FGN, and accurate diagnosis requires an integrated approach combining histopathology, immunostaining, and proteomic analysis. Finally, clone-directed therapy targeting the underlying plasma cell disorder may be an effective treatment strategy in monoclonal immunoglobulin–associated renal disease.

## Supplementary Information

Below is the link to the electronic supplementary material.

Supplementary Material 1

## Data Availability

Anonymized data supporting the findings of this report are available from the corresponding author upon reasonable request.

## Abbreviations

AHL: Combined heavy- and light-chain amyloidosis
AH: Heavy-chain amyloidosis
AL: Light-chain amyloidosis
AA: Serum amyloid A amyloidosis
ApoE: Apolipoprotein E
BNP: B-type natriuretic peptide
BMI: Body mass index
CH50: 50% hemolytic complement activity
DNAJB9: Dna J heat-shock protein family B member 9
eGFR: Estimated glomerular filtration rate
FGN: Fibrillary glomerulonephritis
GBM: Glomerular basement membrane
ISA: International Society of Amyloidosis
κ: Kappa (light chain)
λ: Lambda (light chain)
LC–MS/MS: Liquid chromatography–tandem mass spectrometry
LMD: Laser microdissection
LMD/MS: Laser microdissection mass spectrometry
MGRS: Monoclonal gammopathy of renal significance
SAP: Serum amyloid P
SAA: Serum amyloid A
