# Supplementary figures and images for: A rare case of renal AHL amyloidosis with marked complement deposition: clinicopathologic and proteomic findings
Source: BMC Nephrol. 2026 Apr 30;27:378. doi: 10.1186/s12882-026-05017-6 (PMC13285500; doi:10.1186/s12882-026-05017-6)

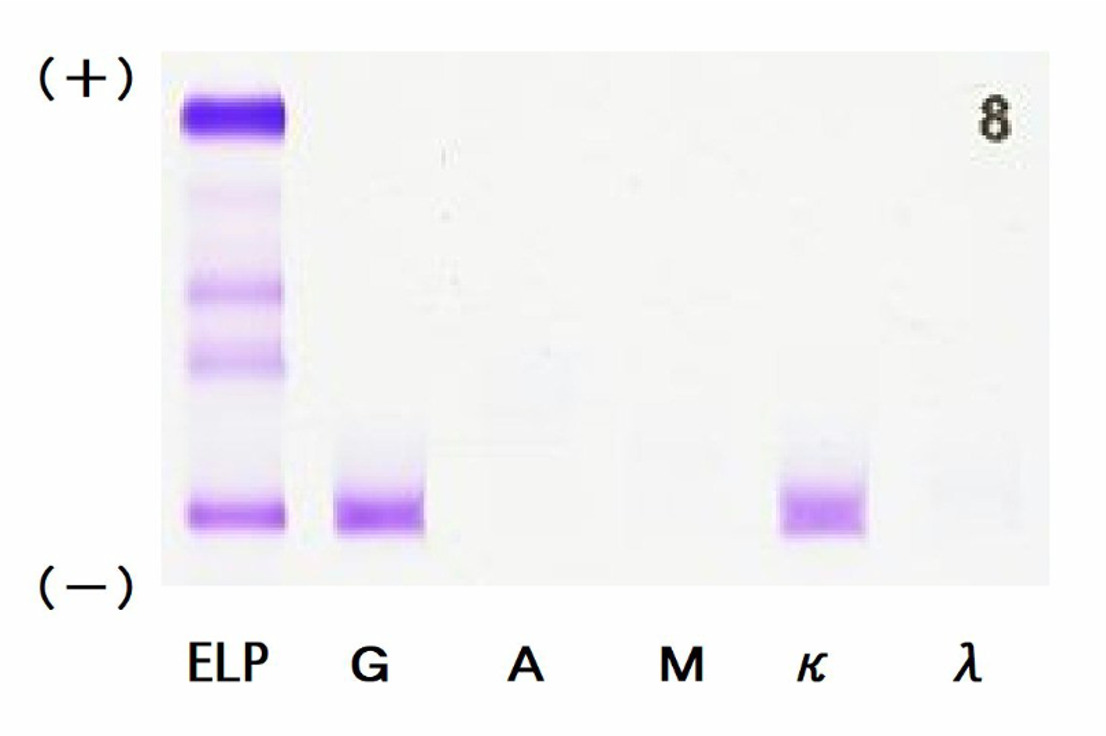

Supplement: Supplementary file 1 — Supplementary Material 1 [file 12882_2026_5017_MOESM1_ESM.png]
